# Isolated Palmar Scaphoid Dislocation: A Case Report and Review of the Literature

**DOI:** 10.7759/cureus.109980

**Published:** 2026-05-31

**Authors:** Lukas Spruyt, Annelien Brauns, Gerry Van der Mieren

**Affiliations:** 1 Department of Emergency Medicine, Ziekenhuis Geel, Geel, BEL; 2 Department of Orthopedic Surgery, Ziekenhuis Geel, Geel, BEL

**Keywords:** case report, isolated scaphoid dislocation, kirschner wire fixation, trauma, wrist injury

## Abstract

Scaphoid dislocations are extremely rare injuries that typically occur in association with carpal fractures or complex carpal dissociation patterns. Isolated scaphoid dislocations, particularly palmar dislocations without associated fractures, are exceptionally uncommon. Early recognition and prompt management are essential to prevent complications such as persistent carpal instability or degenerative changes.

We report the case of a 47-year-old female who sustained an isolated palmar dislocation of the scaphoid following a fall onto the ulnar side of the left wrist. Initial plain radiography suggested abnormal scaphoid alignment without fracture; computed tomography confirmed an isolated palmar scaphoid dislocation. Closed reduction under locoregional anesthesia was achieved by applying palmar-to-dorsal directed pressure on the scaphoid tubercle. Percutaneous Kirschner wire (K-wire) fixation was subsequently performed to maintain reduction. Immobilization in an open scaphoid cast was maintained for six weeks.

After six weeks, the K-wires were removed and the patient started physiotherapy. This case highlights the importance of recognizing this rare injury pattern and supports early reduction with temporary fixation as an effective management strategy. A brief review of the literature is provided.

## Introduction

Scaphoid dislocations represent an uncommon pattern within carpal injuries and are typically associated with additional fractures or perilunate dislocations. Isolated dislocation without concomitant fractures or perilunate involvement is extremely rare, with only a small number of cases documented in the literature since the first report by Higgs in 1930 [1].

Diagnosis can be challenging on initial plain radiography because the injury often results primarily from ligamentous disruption rather than obvious osseous injury. When clinical suspicion persists, computed tomography (CT) imaging may aid in confirming the diagnosis and assessing carpal alignment. Early reduction and stabilization aim to restore carpal alignment and prevent complications such as instability, degenerative arthritis, or avascular necrosis [2].

Multiple case reports and reviews have explored various strategies ranging from closed reduction with immobilization to open reduction with ligamentous repair and fixation [2,3].

## Case presentation

A 47-year-old right-hand dominant female presented to the emergency department after sustaining a fall in her garden. She reported falling onto her outstretched left hand, with the wrist in ulnar deviation at the moment of impact. She immediately experienced severe wrist pain, swelling, and an inability to actively move the wrist.

Clinical examination demonstrated a fixed deformity of the left wrist, characterized by radial deviation and extension. Mild swelling and ulnar-sided ecchymosis were present, without any open wounds. Palpation elicited diffuse tenderness over the carpal region, particularly over the radial aspect of the wrist. Active range of motion was severely limited due to pain, while passive motion was also restricted. Neurovascular examination was unremarkable, with intact sensation, preserved capillary refill, and maintained finger flexion and extension (Figure 1).

**Figure 1:**
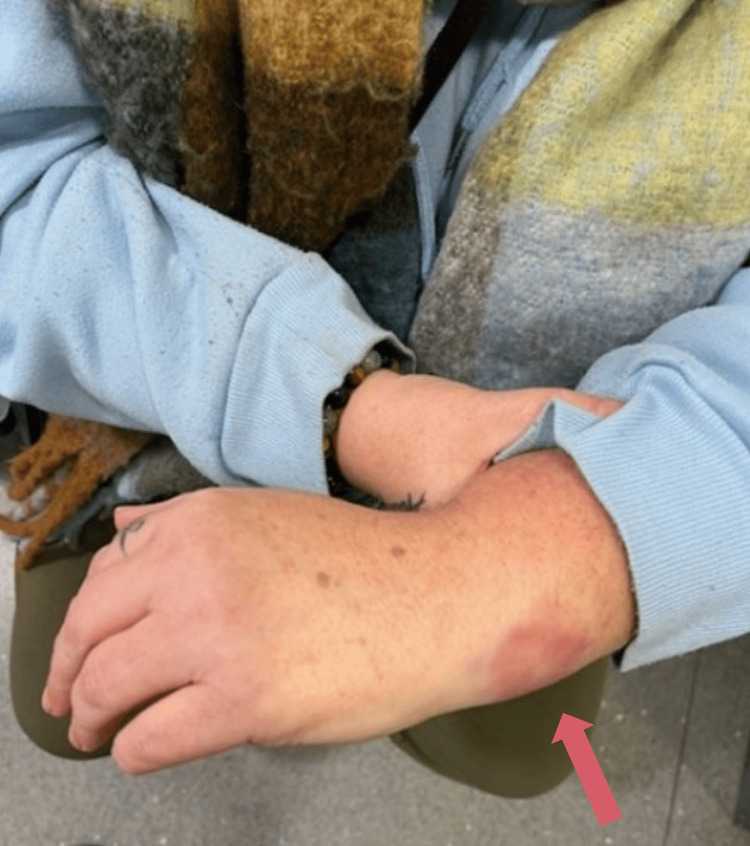
Clinical photograph obtained in the emergency department showing the injured left wrist following trauma.

Initial plain radiography of the wrist did not reveal any fractures but raised suspicion of carpal malalignment, particularly involving the scaphoid (Figure 2).

**Figure 2:**
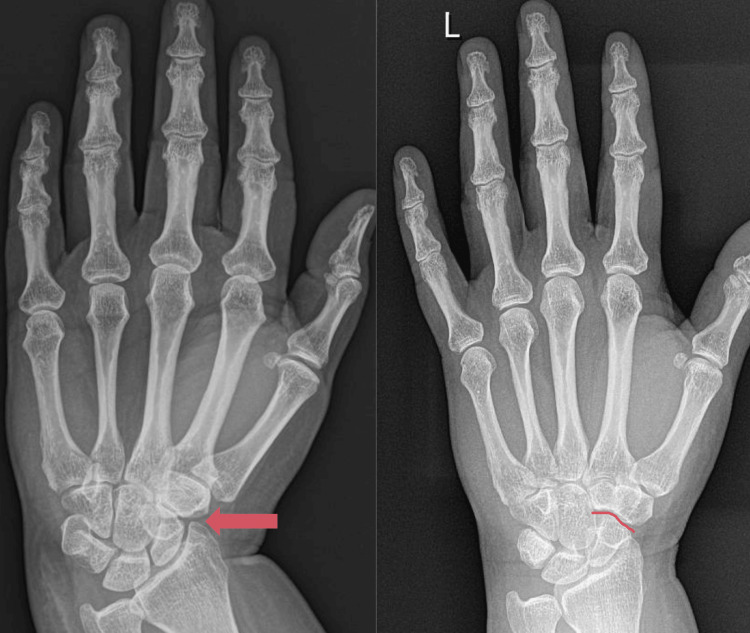
Anteroposterior radiography of the left wrist. Left side: the normal scaphotrapeziotrapezoidal (STT) joint can no longer be aligned and the carpals are projecting onto each other. Right side: the normal alignment of the STT joint.

Given the inconclusive nature of the radiographs and concern for a complex carpal injury, including possible perilunate instability or isolated carpal dislocation, a CT scan was performed. CT imaging confirmed an isolated palmar dislocation of the scaphoid, without associated fractures or evidence of perilunate dislocation (Figures 3, 4).

**Figure 3:**
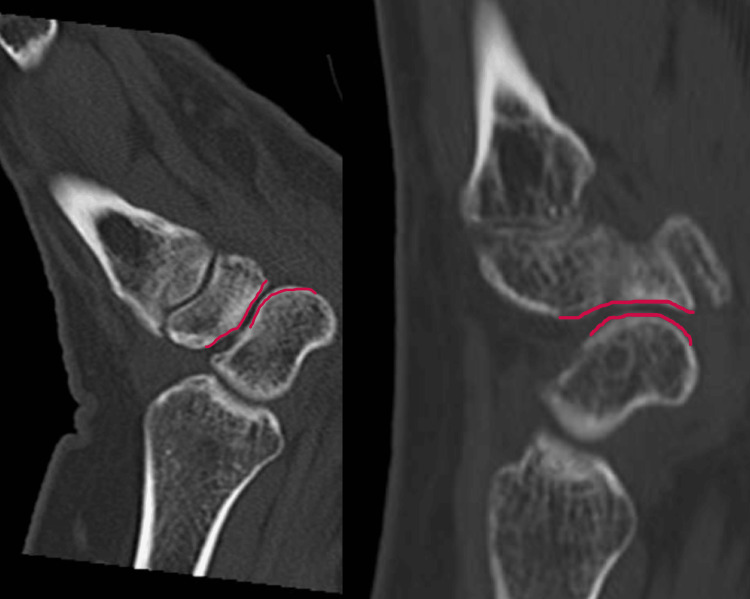
CT scan, sagittal view. The articulating scaphotrapezoid joint surface is marked in red. Left side: preoperative image showing the dislocation. Right side: postoperative normal view after removal of K-wires

**Figure 4:**
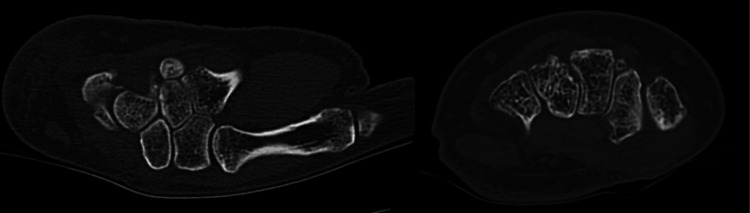
CT scan, axial view. Left side: preoperative view in which the scaphoid and trapezium project in one plane. Right side: postoperative view in which the trapezoid lies back more distally than the scaphoid

Closed reduction was performed under locoregional anesthesia. Longitudinal traction combined with gentle manipulation resulted in a palpable and audible “clunk,” indicating successful reduction. Post-reduction clinical assessment demonstrated improved wrist alignment and increased range of motion compared to the pre-reduction status. No residual tendency toward radial deviation was observed. Fluoroscopic evaluation confirmed anatomic reduction and apparent stability of the scaphoid.

However, given the recognized risk of recurrent instability in isolated scaphoid dislocations, supplementary fixation was performed. Two Kirschner wires (K-wires) were inserted percutaneously: one between the scaphoid and trapezium and one between the scaphoid and capitate (Figure 5).

**Figure 5:**
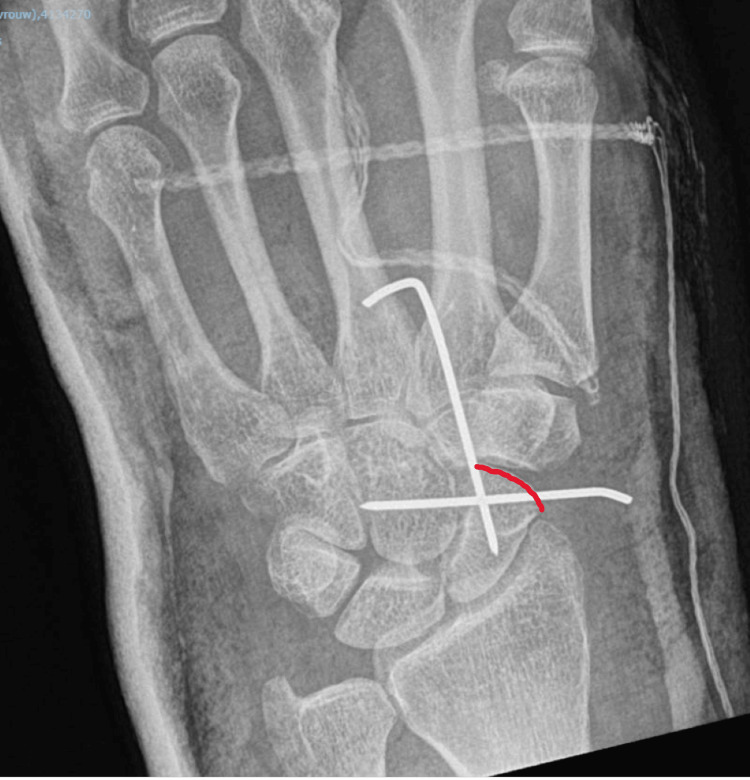
Post-reduction radiography of the left wrist demonstrating the restored alignement of the scaphotrapeziotrapezoidal (STT) joint.

Following fixation, immobilization was applied using a scaphoid cast, which was maintained for a total duration of six weeks.

After six weeks, the K-wires were removed and the cast was discontinued. A removable wrist brace was initiated immediately after cast removal. At clinical follow-up two weeks later, the wrist demonstrated a good active and passive range of motion. The patient reported no pain during wrist mobilization, including flexion, extension, and radial and ulnar deviation. However, she continued to experience localized pain in the region of the first dorsal compartment (de Quervain region), particularly during thumb movement, described as a dull, aching pain.

Following this evaluation, a structured physiotherapy program focusing on gradual mobilization and strengthening was initiated. The patient was advised to continue wearing the wrist brace with progressive weaning.

## Discussion

Isolated scaphoid dislocations represent a rare form of carpal injury compared with more common patterns such as perilunate dislocations or scaphoid fractures. Literature reviews estimate that approximately 48 to 55 cases of isolated scaphoid dislocation have been reported in the English-language literature to date [2,3]. The injury is thought to result from a combination of axial loading, wrist dorsiflexion, and ulnar deviation, producing progressive disruption of the stabilizing carpal ligaments.

Several classification systems have been proposed to better characterize these injuries. Leung et al. described a classification based on four parameters: primary versus secondary dislocation, simple versus complex injury patterns, partial versus total dislocation, and the direction of displacement [4]. Most reported cases involve partial and palmar dislocations, consistent with the mechanism of ligamentous failure affecting the radioscaphocapitate and scaphotrapezial stabilizing structures.

Diagnosis can be challenging on initial plain radiography because these injuries are primarily ligamentous and may occur without associated fractures. Subtle malalignment of the scaphoid may therefore be overlooked on plain radiographs. CT can provide additional detail regarding carpal alignment and help confirm the diagnosis, particularly in cases of palmar displacement, as in the present case.

Early recognition is important because delayed diagnosis has been associated with a higher likelihood of requiring open reduction and potentially poorer functional outcomes [2,3]. Various management strategies have been described in the literature. Closed reduction followed by immobilization or temporary K-wire fixation is often successful when the injury is diagnosed early and the dislocation is partial [3]. In contrast, open reduction may be required in cases of total dislocation, failed closed reduction, or when soft-tissue interposition prevents stable reduction. Some authors advocate additional ligament repair in these situations to restore carpal stability [3].

More recently, arthroscopy-assisted reduction has also been described as a minimally invasive technique that allows direct visualization of the carpal joints and associated ligamentous injuries [3]. However, due to the rarity of this injury pattern, no standardized treatment algorithm exists and management decisions are often based on individual case characteristics and surgeon preference.

In the present case, early diagnosis allowed successful closed reduction followed by percutaneous K-wire fixation and immobilization. This approach is consistent with previously reported cases in which early management resulted in satisfactory functional outcomes [2,3]. Reported complications of isolated scaphoid dislocations include scapholunate diastasis, chronic carpal instability, degenerative joint changes, and, rarely, avascular necrosis of the scaphoid [2,3,5]. Prompt restoration of carpal alignment is therefore essential to minimize the risk of long-term morbidity. Due to the rarity of this injury pattern, each additional reported case contributes valuable information regarding diagnosis and optimal management strategies.

## Conclusions

Isolated palmar scaphoid dislocation is a rare clinical entity. In case of clinical suspicion, liberal use of CT imaging is crucial to confirm diagnosis. Appropriate reduction with stabilization, whether closed or open with fixation, contribute to favorable outcomes. Awareness of this injury pattern, together with careful review of the existing literature, may assist clinicians in effective management planning.
